# Size-Dependent Antibacterial, Antidiabetic, and Toxicity of Silver Nanoparticles Synthesized Using Solvent Extraction of *Rosa indica* L. Petals

**DOI:** 10.3390/ph15060689

**Published:** 2022-05-31

**Authors:** Satheesh Kumar Balu, Swetha Andra, Fouad Damiri, Anandhi Sivaramalingam, Manisha Vidyavathy Sudandaradoss, Karthikeyan Kumarasamy, Kishore Bhakthavachalam, Faraat Ali, Milton Kumar Kundu, Md. Habibur Rahman, Mohammed Berrada, Simona Cavalu

**Affiliations:** 1 Department of Oral Pathology, Saveetha Dental College and Hospitals, Saveetha Institute of Medical and Technical Sciences, Chennai 600077, India; bsatheeshsk@gmail.com; 2 Centre for Nanoscience and Technology, Chennai Institute of Technology, Chennai 600069, India; swethavenkatesh3891@gmail.com; 3 Laboratory of Biomolecules and Organic Synthesis (BIOSYNTHO), Department of Chemistry, Faculty of Sciences Ben M’Sick, University Hassan II of Casablanca, Casablanca 20000, Morocco; fouad.damiri@outlook.fr (F.D.); berrada_moh@hotmail.com (M.B.); 4 Department of Physics, Sathyabama Institute of Science and Technology, Chennai 600119, India; kishoreabi1309@gmail.com; 5 Department of Ceramic Technology, Anna University, Chennai 600025, India; manisha@annauniv.edu (M.V.S.); karthiken96@gmail.com (K.K.); 6 Department of Licensing and Enforcement, Laboratory Services, Botswana Medicines Regulatory Authority (BoMRA), Gaborone 999106, Botswana; frhtl6@gmail.com; 7 Pharmacy Department, Khulna University, Khulna 9208, Bangladesh; miltonkundu@gmail.com; 8 Department of Global Medical Science, Wonju College of Medicine, Yonsei University, Wonju 26426, Korea; pharmacisthabib@gmail.com; 9 Faculty of Medicine and Pharmacy, University of Oradea, P-ta 1 Decembrie 10, 410087 Oradea, Romania

**Keywords:** silver nanoparticles, *Rosa indica* L. extracts, antibacterial, antidiabetic, antioxidants

## Abstract

In this study, silver nanoparticles (AgNPs) are synthesized through a green approach by employing *Rosa indica* L. petal (RE) extracts as reducing and stabilizing agents, which are extracted using three different solvents: ethanol (Et), acetone (Ac), and water (Aq). The phase formation of the AgNPs is confirmed using X-ray diffraction (XRD). Morphological analysis is performed using a field-emission scanning electron microscope (FESEM), which reveals that the AgNPs are spherical in shape. The size is estimated using ImageJ software, which is found to be ~12, 18, and 770 nm for RE-Ac-Ag, RE-Et-Ag, and RE-Aq-Ag, respectively. The phytochemicals of *Rosa indica* L. petals involved in the formation of the AgNPs are studied using Fourier transform infrared spectroscopy (FTIR). Finally, these materials are studied for their antibacterial, antidiabetic, antioxidant, and hemolytic activity, as well as cell toxicity properties. The materials, RE-Ac-Ag and RE-Et-Ag, are found to be more effective than RE-Aq-Ag in inhibiting *E. coli* (Gram-negative bacteria) and *S. aureus* (Gram-positive bacteria). Hemolytic studies reveal that all of the samples show concentration-dependent activity up to 50 µg/mL. RE-Ac-Ag and RE-Et-Ag exhibit nonhemolytic behavior, whereas RE-Aq-Ag remains nonhemolytic until 100 µg/mL. The antidiabetic ability of the AgNPs is evaluated using α-amylase inhibition assay (DNSA assay) and α-glucosidase inhibition assay. The results are found to be effective, with IC_50_ values of α-amylase and α-glycosidase being 50, 50, and 75 µg/mL for RE-Et-Ag, RE-Ac-Ag, and RE-Aq-Ag, respectively. DPPH assay shows that the AgNPs inhibited the antioxidants well, with IC_50_ values of 40 µg/mL for RE-Et-Ag and RE-Ac-Ag and 60 µg/mL for RE-Aq-Ag. The toxicity study reveals that the AgNPs show size- and concentration-dependent behavior. Overall, it is realized from the findings that RE-Ac-Ag, RE-Et-Ag, and RE-Aq-Ag show size-dependent antibacterial, antidiabetic, and toxicity properties.

## 1. Introduction

The widespread use of nanoparticles in medicine, technology development, and industry is increasing the demand for controlled synthesis of nanomaterials with improved multifunctional properties. Among the metallic nanomaterials, silver nanoparticles (AgNPs) are frequently used in photocatalysis, antimicrobial treatments, microelectronics, and photonics applications [1,2]. Nanomedicine is a thriving research area. Scientists are concerned about developing safe, efficient, and less hazardous drugs to treat diseases such as epilepsy, cancer, and diabetes [3,4]. The fast clearance rate of silver nanoparticles from the body is one of the benefits of using them in biomedical applications [5,6]. Microbial infections have recently become a major concern in the healthcare industry [7,8,9], particularly in the case of dental and orthopedic implants. Microbial formation on implantable devices can cause wound healing to be delayed and eventually lead to implant failure and require implant replacement [10,11].

According to a recent study, combining silver nanoparticles with filler materials improves root canal activity when compared to existing root canal therapies [12]. Microgalvanic effects occur when titanium (Ti) is coated with AgNPs, enhancing antibacterial activity and osteoblast compatibility. Biological approaches have been often used over chemical procedures to synthesize AgNPs since chemical methods involve the use of toxic reducing agents that are dangerous to both humans and the environment. Fungi, bacteria, and plants are used in the biological synthesis of nanoparticles (NPs) [13]. However, due to specialized requirements during maintenance, the use of bacteria and fungi in the production of nanomaterials is limited. 

As a result, plant-based biosynthesis has emerged as a promising alternative to the chemical approach for reducing the risk of hazardous chemicals and their inflammatory effects [14]. Several studies have discovered that Gram-negative bacteria have developed resistance to AgNPs because of the development of flagellin proteins, which lower the antibacterial activity of AgNPs. As a result, combining silver nanoparticles with phytochemical ingredients can prevent the aforementioned problems [15]. Antioxidants are essential in maintaining a healthy balance of free radicals, as these free radicals attack macromolecules, such as proteins, nucleic acids, and lipids, resulting in cell damage and death. Chronic abnormalities such as heart disease, the development of cancer [16], and neurodegenerative disorders are exacerbated by the increased accumulation of reactive oxygen species in the environment. Despite the fact that the literature indicates that AgNPs have significant activity in vitro, their therapeutic efficacy is still not fully determined [17]. Rose petals are rich sources of anthocyanins, which are phenolic compounds that belong to the flavonoids family and have two benzene rings connected by a three-carbon linear chain with the basic skeleton C6–C3–C6 associated with sugar molecules. Traditionally, anthocyanins have been utilized as natural food colorants. The strong antioxidant property of anthocyanins helps prevent cardiovascular illness, diabetes, cancer, neuronal diseases, ulcers, and inflammation [18]. Accordingly, *Rosa indica* L. petals could be a suitable phytochemical source to prepare AgNPs.

In this context, the present study demonstrates the successful synthesis of AgNPs using *Rosa* *indica* L. petals extracted using ethanol, acetone, and water, and the effects of these solvents on the properties of AgNPs are compared. Furthermore, these materials are also examined for their efficacy towards antibacterial, antidiabetic, antioxidant, hemolytic activity, and cell toxicity properties.

## 2. Results and Discussion 

### 2.1. X-ray Diffraction (XRD)

**Figure 1:**
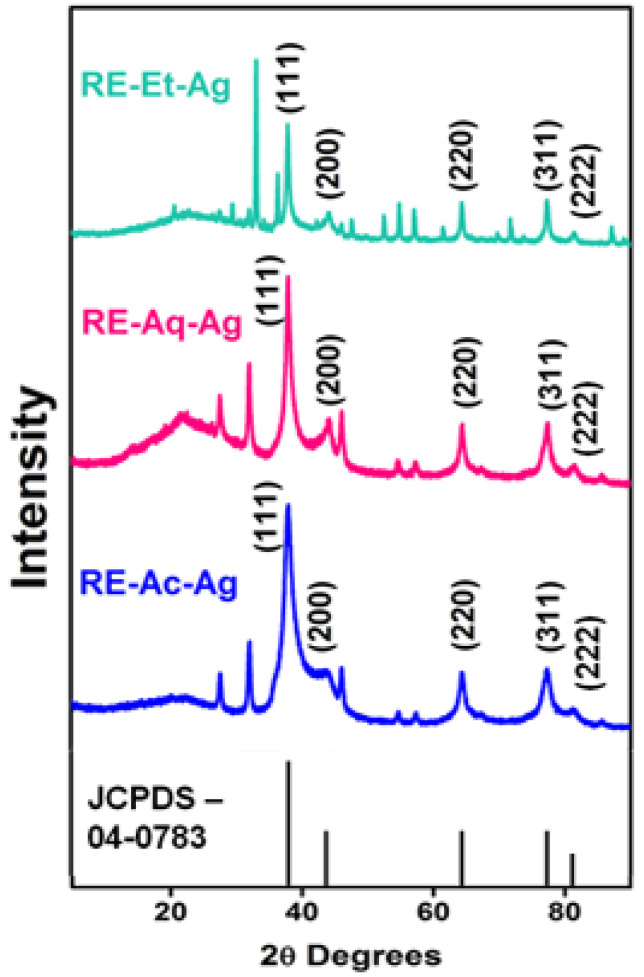
XRD patterns of RE-Ac-Ag, RE-Aq-Ag, and RE-Et-Ag nanoparticles.

As shown in Figure 1, the XRD patterns of the phytosynthesized AgNPs are very similar to those of the standard AgNPs (JCPDS No.: 89-3722). The diffraction peaks are detected at 37.9°, 43.9°, 64.3°, and 77.3°, which correspond to the (111), (200), (220), and (311) planes, respectively, indicating that the AgNPs have a face-centered cubic (FCC) structure. Peaks appearing at 27.7° and 31.9° can be attributed to the deposition of phytocompounds by RE. The crystallite sizes of the prepared AgNPs were calculated using Debye–Scherrer’s formula, and the average crystallite is estimated to be 14, 17, and 26 nm for RE-Ac-Ag, RE-Et-Ag, and RE-Aq-Ag, respectively. The result suggests that the crystallite size of the AgNPs increased with respect to the various biomolecules present in the solvents.

### 2.2. Functional Group Analysis

**Figure 2:**
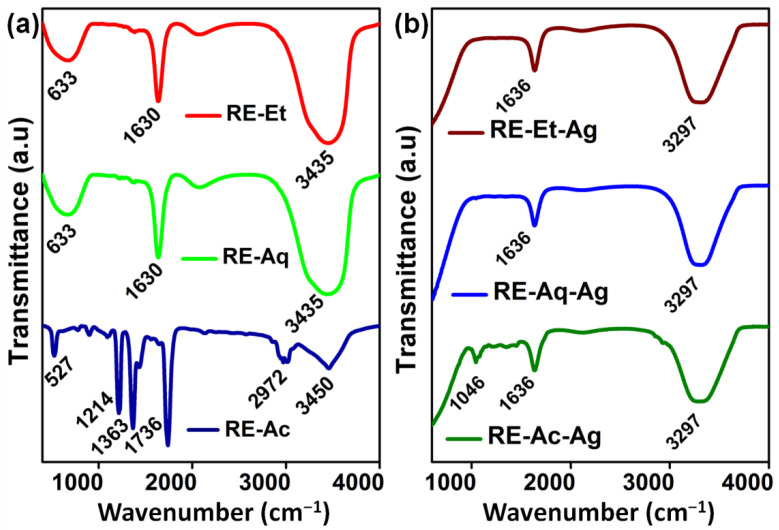
FTIR spectrum of plant extracts: (**a**) RE-Ac, RE-Aq, and RE-Et; (**b**) AgNPs RE-Ac-Ag, RE-Aq-Ag, and RE-Et-Ag.

Fourier transform infrared spectroscopy (FTIR) measurements were used to detect the functional groups that were present in the *Rosa indica* L. extract, which is responsible for the reduction process of Ag^+^ ions and stabilization of AgNPs. In the spectra of RE-Et, RE-Aq, and RE-Ac, shown in Figure 2a, the bands at 3435 and 3450 cm^−1^ correspond to the O-H stretching vibration, suggesting the presence of phenol and alcohol. The band at 2972 cm^−1^ in RE-Ac is formed by aromatic compound C-H stretching, but this peak is not found in RE-Et or RE-Aq. The C=O stretching vibrations are allocated to the band at 1630 cm^−1^ in RE-Et and RE-Aq. The band appearing at 633 cm^−1^ in RE-Et and RE-Aq indicates C-C stretching in the alkyl group. The peaks at 1736 cm^−1^, 1363 cm^−1^, and 1214 cm^−1^ in RE-Ac can be ascribed to C-C stretching, N=O stretching of nitro compounds, and C-N stretching of amines, respectively [19,20]. The existence of bands at 3297, 1636, and 1046 cm^−1^ for RE-Et-Ag, RE-Aq-Ag, and RE-Ac-Ag is shown in Figure 2b. These FTIR findings clearly indicate that several biological molecules are likely to be involved in the synthesis and stabilization of AgNPs [21]. Furthermore, slight peak shifts are observed for all the samples, and several peaks disappeared in RE-Ac-Ag, which suggests that the biomolecules in the extracts are involved in the formation of AgNPs.

### 2.3. Field-Emission Scanning Electron Microscopy (FESEM)

**Figure 3:**
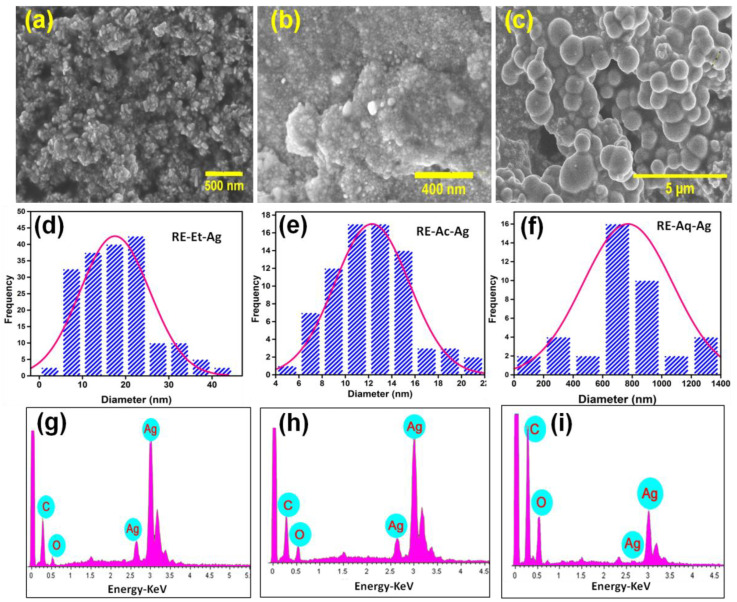
FESEM micrographs of AgNPs: (**a**) RE-Et-Ag, (**b**) RE-Ac-Ag, and (**c**) RE-Aq-Ag. (**d**–**f**) Average particle size of RE-Et-Ag, RE-Ac-Ag, and RE-Aq-Ag. (**g**–**i**) EDS spectrum of RE-Et-Ag, RE-Ac-Ag, and RE-Aq-Ag.

The morphological structure is an important indicator because it can directly impact the electro-optic and biological properties. Figure 3a–c displays the FESEM images showing the spherical-like morphology of the AgNPs. Figure 3d–f reveals the average size of the three samples, RE-Et-Ag, RE-Ac-Ag, and RE-Aq-Ag, is estimated to be around 18, 12, and 770 nm, respectively. The solvents employed in the fabrication of silver nanoparticles clearly altered the size of the nanoparticles. The shape of the formed AgNPs does not vary considerably with size; therefore, the discrepancies in antibacterial outcomes can only be ascribed to changes in the size of the nanoparticles [1,22]. Similarly, Jahan et al. (2019) prepared AgNPs using *Rosa santana* extract in microwave conditions, and transmission electron microscope (TEM) results showed spherical-shaped NPs with an average size of 14 nm [23]. Suarez-cerda et al. (2015) showed that AgNPs size depends on the concentration of *Rosa* extracts [24]. *Hibiscus rosa-sinensis* extract was used as a reducing and capping agent for AgNP synthesis. The SEM results showed an aggregated spherical-shaped morphology with an average size of ~48.5 nm [25]. These results showed the potential synthesis of AgNPs using aqueous extracts of plants. Furthermore, as shown in Figure 3g–i, the energy-dispersive X-ray spectroscopy (EDS) spectrum of the green-produced AgNPs offered semiquantitative information on the elemental content of the samples. The EDS spectra clearly show that the rose extracts successfully participated in the formation of AgNPs. Furthermore, RE-Aq-Ag (Figure 3i) contains less Ag% than RE-Et-Ag and RE-Ac-Ag, which may influence its biological properties.

### 2.4. Antimicrobial Activity

**Figure 4:**
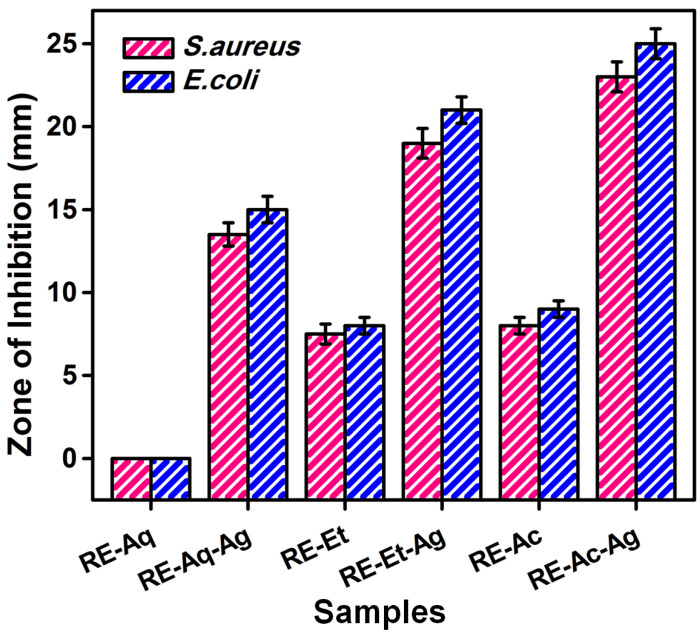
Antibacterial activities of *Rosa indica* L. pedal extracts and silver nanoparticles (values with mean + SD, n = 3).

AgNPs with different sizes were tested by the well diffusion method for their inhibitory effect against *E. coli* and *S. aureus* bacterial strains. The antibacterial experiments reveal that all three samples have strong antibacterial activity against both bacteria; however, the obtained zone of inhibition values showed size-dependent activity, as shown in Figure 4. Additionally, the lower amount of Ag in the sample RE-Aq-Ag may have affected its bactericidal ability. These findings reveal that the smaller-sized AgNPs obtained from the solvent acetone (RE-Ac-Ag) and ethanol (RE-Et-Ag) have greater inhibitory efficacy than the water-derived AgNPs (RE-Aq-Ag). The current study demonstrated that AgNPs with sizes of 12 nm (RE-Ac-Ag) and 18 nm (RE-Et-Ag) have zone of inhibition values of 25 and 21 mm against *E. coli* and 23 and 19 mm against *S. aureus*, respectively. In the case of larger-sized (770 nm) AgNPs (RE-Aq-Ag), the antibacterial activity against *E. coli* and *S. aureus* is determined to be 15 and 13.5 mm, respectively. The observed changes in the antibacterial activity can be attributed to the size variation of the nanoparticles and the percentage of Ag, as well as the abundant availability of the functional groups of RE molecules extracted using acetone and ethanol, which are found to be responsible. In addition, the phytochemicals facilitated the ability to produce more reactive oxygen species (ROS), which are then used to inhibit bacterial growth [26]. This also demonstrated that the effect of nonaqueous solvents on the surface chemistry of the AgNPs produced improved their antibacterial capabilities by allowing them to readily interact with the bacterial nucleus, resulting in the highest interaction of the nanoparticles with the bacteria for efficient killing. For instance, Loo et al. (2018) synthesized AgNPs using aqueous extracted tea leaves, and the antibacterial activity against *E. coli* was found to be 15 mm. Likewise, Ahmed et al. (2016) synthesized AgNPs using aqueous leaf extract of *Azadirachta indica*. The antibacterial activity results against *E. coli* and *S. aureus* were determined to be 9 mm for both bacterial strains [19]. Previous literature has also suggested that nanoparticles exhibit size-dependent antibacterial action. Smaller particles have shown stronger antibacterial action due to their better capacity to penetrate bacteria. Smaller AgNPs have a superior surface area than larger bacteria, resulting in an increased antibacterial action. The results showed that AgNPs are more harmful to *E. coli* than to *S. aureus*. This might be related to changes in the rate of diffusion, structure of the cell, cell metabolism, and surface interaction of nanoparticles with microorganisms [27].

### 2.5. Hemolysis

**Figure 5:**
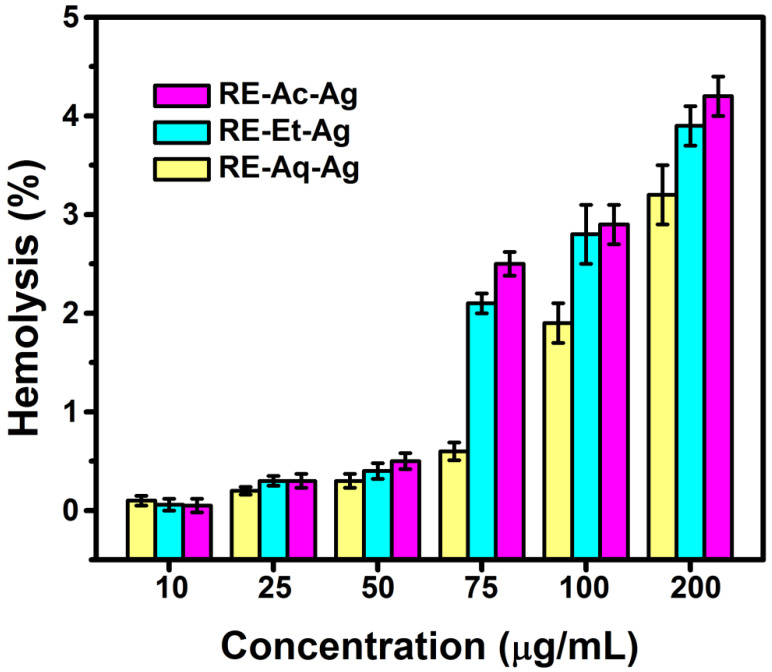
Hemolysis results of green synthesized silver nanoparticles (values with mean + SD, n = 3).

The size-dependent hemolytic behavior of AgNPs was evaluated at concentrations ranging from 10 to 200 µg/mL (Figure 5). From the results, it is observed that the samples RE-Et-Ag and RE-Ac-Ag demonstrated less than 2% hemolysis until 50 µg/mL, indicating that the materials are totally nonhemolytic. However, at concentrations from 75 µg/mL to 200 µg/mL, the hemolytic percentage is around 2 to 5%, indicating that the materials show slight hemolytic action at these doses. The sample RE-Aq-Ag is found to be nonhemolytic up to 100 µg/mL, but at 200 µg/mL, it displays mildly hemolytic activity. The results show that AgNPs with smaller sizes had higher hemolytic activity than AgNPs with larger sizes. Further, the results obtained suggest that the hemolytic effect is concentration dependent. According to the literature, smaller nanoparticles are more harmful than the bigger particles because they can readily breach the membrane due to their larger surface area and may powerfully interact with biomolecules [28,29]. Chen et al. (2015) observed size-dependent hemolytic activity of AgNPs with sizes of 15, 50, and 100 nm against fish RBCs. The results show that nanoparticles with a size of ~15 nm have a stronger capacity to cause hemolysis and membrane damage than nanoparticles with sizes around 50 and 100 nm. It can be stated that such hemolytic behavior caused by AgNPs should be ascribed to the nanoparticle’s direct contact with the RBCs, which results in the generation of oxidative stress, membrane damage, and, ultimately, hemolysis. According to the findings, the particle size of AgNPs has a significant impact during their interaction with RBCs [30].

### 2.6. Cell Viability

**Figure 6:**
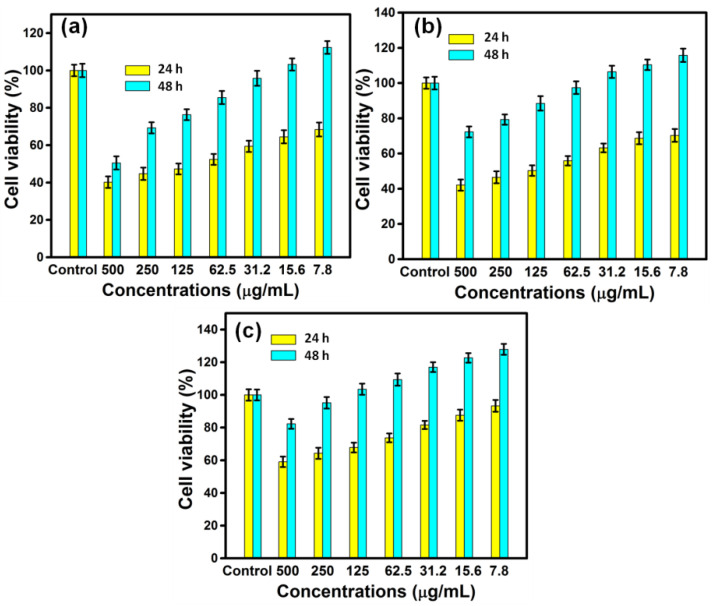
Biocompatibility results of (**a**) RE-Ac-Ag, (**b**) RE-Et-Ag, and (**c**) RE-Aq-Ag (values with mean + SD, n = 3).

**Figure 7:**
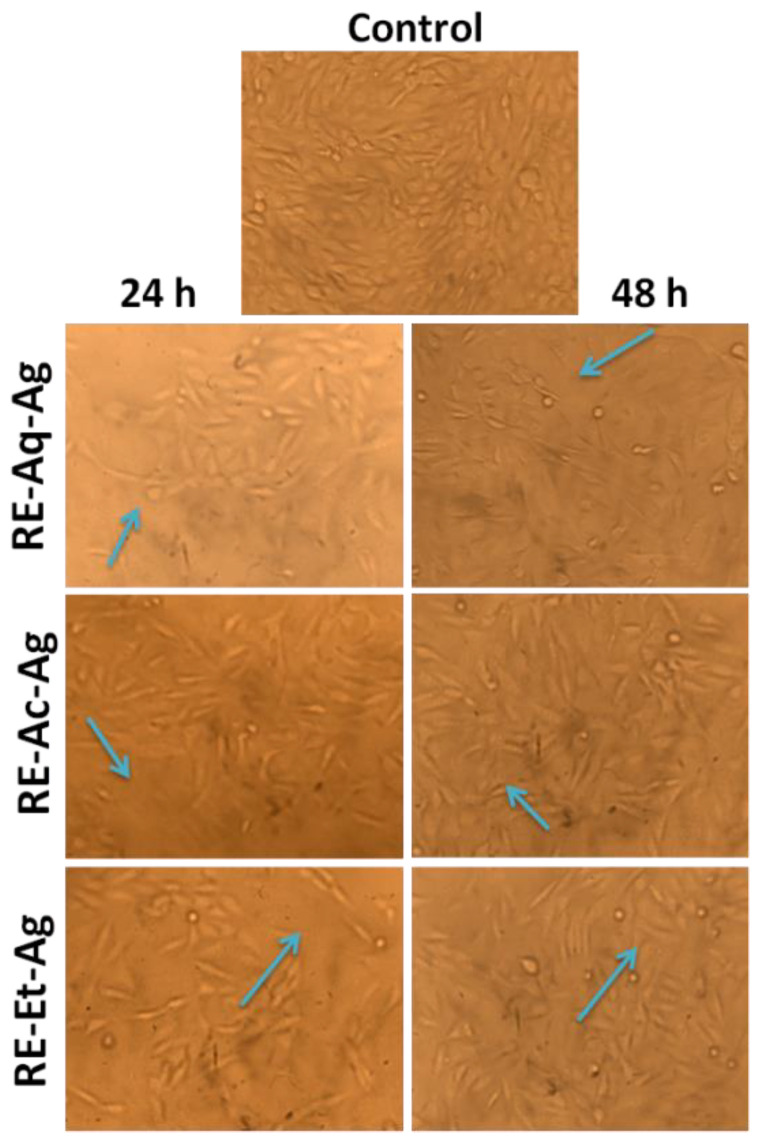
Optical microscopic images MG63 cell lines treated with RE-Ac-Ag, RE-Et-Ag, and RE-Aq-Ag.

MTT assay was carried out to assess the influence of AgNPs on the proliferation rate of MG63 cell lines (Figure 6). The cells were cultured for 24 and 48 h with varied sizes of AgNPs at varying concentrations, and the viability of cells was determined by plotting the graph of cell proliferation efficiency. The findings clearly demonstrated the size-dependent cytotoxic activity, with the samples RE-Et-Ag and RE-Ac-Ag exhibiting cytotoxic behavior at 125 µg/mL and RE-Aq-Ag exhibiting cytotoxic behavior at 500 µg/mL. Furthermore, the obtained data also revealed that the AgNPs behave in a concentration-dependent way; as the concentration increased from 7.8 to 500 µg/mL, the viability of all samples reduced. Cell viability dropped after 24 h of incubation for the samples RE-Et-Ag and RE-Ac-Ag. In contrast, after 48 h of incubation, the cell proliferation rate in lower concentrations increased above the control. This might be attributable to AgNPs achieving the optimal conditions after extending the incubation time, which increased the rate of cell growth [31]. Optical microscopic images (Figure 7) also revealed similar results, demonstrating that cell proliferation is visible after 48 h as compared to 24 h. Soares et al. (2016) studied the size-dependent cytotoxicity of AgNPs using the sizes of 10 and 50 nm. According to their findings, 10 nm AgNPs are more hazardous than 50 nm AgNPs. The data showed that nanoparticles impede lysosomal function, cause membrane damage, and cause neutrophils to undergo an oxidative burst. Wu et al. (2019) investigated the cytotoxicity of nanoparticles against B16 cells of different sizes, finding that nanoparticles as small as 5 nm were found in the cytoplasm and nucleus, whereas the larger nanoparticles (20, 50, and 100 nm) were found outside the cells. This might be owing to the ease with which smaller nanoparticles penetrated the cells, but bigger nanoparticles were exceedingly difficult to enter [32].

### 2.7. Inhibitory Effect of α-Amylase and α-Glucosidase

**Figure 8:**
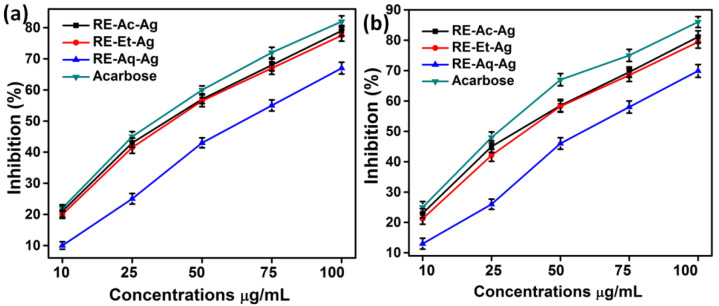
(**a**) Inhibitory effect of α-amylase and (**b**) α-glucosidase at various concentrations of AgNPs (values with mean + SD, n = 3).

Alpha-amylase (α-amylase) and alpha-glucosidase (α-glucosidase) are enzymes that contribute to carbohydrate breakdown and glucose absorption. Inhibiting these two digestive enzymes suppresses starch breakdown and slows down the glucose release in the blood. AgNPs were incubated with α-amylase and α-glucosidase at various concentrations (10, 25, 50, 75, and 100 µg/mL), and acarbose was used as a control. Figure 8a,b shows that α-amylase and α-glucosidase were significantly inhibited in a dose-dependent manner. The percentage of inhibition is found to be increased with the increasing concentration of AgNPs. The IC_50_ values of α-amylase and α-glucosidase are determined to be around 50, 50, and 75 µg/mL for RE-Et-Ag, RE-Ac-Ag, and RE-Aq-Ag respectively. Thus, the results indicated that the AgNPs and acarbose show a significant inhibition of α-amylase and α-glucosidase. Likewise, Balan et al. (2016) reported the antidiabetic activity of AgNPs, with IC_50_ values of 54.56 µg/mL and 37.86 µg/mL for α-amylase and α-glucosidase, respectively. In another study, Saratalae et al. (2018) developed AgNPs using *Punica granatum* leaves and tested them for their antidiabetic activity. The IC_50_ values were found to be 65.2 and 53.8 µg/mL for α-amylase and α-glucosidase, respectively [17].

### 2.8. DPPH Assay

**Figure 9:**
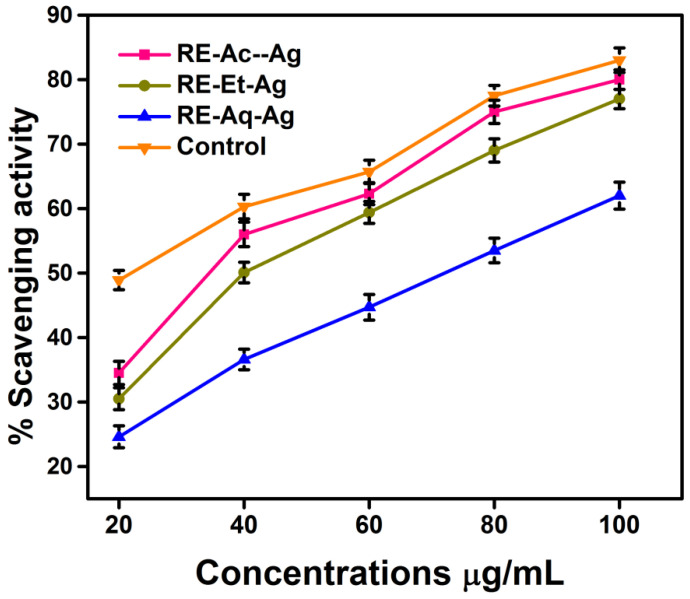
DPPH radical scavenging activity prepared AgNPs (values with mean + SD, n = 3).

The capacity to scavenge free radicals is examined using the free radical compound DPPH. The scavenging ability is determined at various concentrations of AgNPs (20, 40, 60, 80, and 100 µg/mL) via the characteristic absorbance at 517 nm. The reduction in absorption indicates that the free radicals are being scavenged [22]. The findings, as depicted in Figure 9, suggested that the free radical scavenging efficacy of RE-Et-Ag, RE-Ac-Ag, and RE-Aq-Ag is size dependent. The IC_50_ values of DPPH inhibition were found to be ~40 µg/mL for RE-Et-Ag and RE-Ac-Ag and 60 µg/mL for RE-Aq-Ag. Additionally, increasing the concentration of the samples enhanced the scavenging efficacy of the AgNPs, as well as the activity of the positive control by 83, 80, 77, and 62% at 100 µg/mL for ascorbic acid, RE-Et-Ag, RE-Ac-Ag, and RE-Aq-Ag, respectively. According to the results, AgNPs with a lower size have a higher potential for scavenging than the larger nanoparticles. This might be related to the increased surface-to-volume ratio of the smaller AgNPs as compared to the larger-sized NPs, which essentially causes the particles to effectively interact and considerably inhibit the free radicals [17]. The lower percentage of Ag in the larger-sized sample could potentially be contributing to the lower DPPH activity. The findings show that smaller AgNPs have substantial antioxidant properties, which is important for biological applications.

## 3. Materials and Methods

### 3.1. Preparation of Rosa indica L. Petals Extract

Fresh rose flowers (*Rosa indica* L.) were purchased from the local flower market in Adyar, Chennai, India. They were then authenticated by taxonomist Dr. Jeyaraman, Professor, Plant Anatomy Research Centre, Chennai, and voucher specimens (Voucher No.: PARC/2022/4806) were deposited in this department. The *Rosa indica* L. petals were plucked and cleaned with distilled water followed by acetone. Cleaned young petals were cut into small pieces. A 20 g amount of the rose petals (without drying) was taken separately in three conical flasks containing distilled water, ethanol, and acetone, which were denoted as RE-Aq, RE-Et, and RE-Ac, respectively. The beakers RE-Et and RE-Ac were kept undisturbed for 90 min for the extraction of phytochemicals. The RE-Aq-Ag beaker alone was heated at 50 °C for 90 min to extract the phytochemicals. The extract was filtered and stored at 5 °C.

### 3.2. Preparation of Silver Nanoparticles (AgNPs)

In this synthesis method, 90 mL of 0.01 M silver nitrate (AgNO_3_) solution was prepared under constant stirring. To this, 10 mL of RE-Et was added drop wise, and the change in color was observed from pale yellow to reddish-brown. The same procedure was carried out with RE-Ac and RE-Aq. Finally, the prepared AgNPs were employed for centrifugation at 6000 rpm for 30 min, followed by drying in an oven at 60 °C for 1 h. The AgNPs prepared using *Rosa* + ethanol, *Rosa* + acetone, and *Rosa* + water were denoted as RE-Et-Ag, RE-Ac-Ag, and RE-Aq-Ag, respectively [33].

### 3.3. Characterization of AgNPs

The crystallinity of the prepared AgNPs was determined using powder X-ray diffraction by a Bruker diffractometer, model D8 Advance. The crystallite size of the prepared AgNPs was determined using Debye–Scherrer’s formula:

(1) $$D=\frac{K\lambda }{\;\beta \cos\theta }$$

where *D* is the crystallite size, *β* is the full width at half maximum (FWHM), λ is the X-ray wavelength (0.154 nm), *θ* is the diffraction angle, K is a constant related to the crystallite shape and is approximately 0.94. The functional groups in the produced samples and extracts were identified using Fourier transform infrared spectroscopy (FTIR, Perkin Elmer spectrometer, model Spectrum One). The samples were scanned over a range of 4000 to 500 cm^−1^. The shapes and sizes of the synthesized nanoparticles were examined using a field-emission scanning electron microscope (FESEM, JEOL Japan).

### 3.4. Antimicrobial Studies

The standard agar well diffusion method was followed to evaluate the antibacterial efficacy of the prepared AgNPs. The bacterial cultures of *E. coli* and *S. aureus* were procured from the National Centre for Microbial Resource (NCMR, Pune). Nutrient agar was used to cultivate the bacterial strains (SRL Chemicals, India). The nutrient agar was sterilized using an autoclave and transferred to a sterile Petri plate, and the cultured bacterial strains were spread on the solidified agar medium. Afterwards, wells were made by puncturing the agar using a sterile micropipette tip. The prepared AgNPs samples were added to the wells at various concentrations, such as 10, 25, 50, 75, and 100 µL. The zone of inhibition was measured after a 24-hour incubation period at 37 °C [34]. 

### 3.5. Hemolysis Assay

The experiments were carried out in accordance with applicable laws and institutional policies and procedures (Ethical Certificate Ref No.: IHEC/SDC/FACULTY/22/OPATH/416). The blood donor gave informed consent before participating in the experiment. A healthy volunteer’s blood was drawn with the assistance of clinicians, and the results were analyzed. To prevent coagulation, 3.2% trisodium citrate was mixed with blood in a ratio of 1:10. Phosphate-buffered saline (PBS) was used to prepare various concentrations of AgNPs (10, 25, 50, 75, 100, and 200 µg/mL), and anticoagulated blood was used as the test specimen. The positive and negative controls were anticoagulated blood with 0.1% sodium carbonate and PBS with anticoagulated blood, respectively. All the samples and controls were incubated for 3 h at 37 °C. The tubes were then centrifuged for 5 min at 2000 rpm. Finally, OD values were recorded at 545 nm [31]. To ensure reproducibility, all experiments were repeated three times. The hemolysis % was determined as follows:

(2) $$\mathrm{Hemolysis}\;\left(\%\right)=\frac{\left(\mathrm{Test}\;\mathrm{sample}\;-\;\mathrm{Negative}\;\mathrm{control}\right)}{\left(\mathrm{Positive}\;\mathrm{control}\;-\;\mathrm{Negative}\;\mathrm{control}\right)}\times 100$$

### 3.6. MTT Assay

The experiment was conducted to examine the cytocompatibility of the prepared AgNPs. MG63 cell lines were obtained from the National Centre for Cell Sciences (NCCS, Pune). The culture was maintained in a humid atmosphere of 50 g/mL CO_2_ at 37 °C using Dulbecco’s modified Eagle’s medium (DMEM) with 10% fetal bovine serum (FBS), penicillin (100 U/mL), and streptomycin (100 g/mL). In 24-well plates, MG63 cells (approximately 1105/well) were seeded and incubated at 37 °C in a CO_2_ atmosphere. The AgNPs prepared were of various concentrations, such as 7.8, 15.6, 31.2, 62.5, 125, 250, 500, and 1000 µg/mL, and incubated for 24, 48, and 72 h at 37 °C. Then, the cells were treated for 4 h with 3-(4,5-dimethylthiazol-2-yl)-2,5-diphenyl tetrazolium bromide (MTT). To dissolve the formazan crystals, 1 mL of dimethyl sulphoxide (DMSO) was added, and OD values were recorded using an ELISA reader (Bio-Rad 680, USA) at 570 nm. The assay was carried out in triplicate, and the percentage of viable cells was estimated using the following formula:

(3) Cell Viability (%)=(A_570 of treated cells)/(A_570 of control cells)×100

The cells that had been treated with various concentrations of AgNPs were fixed with 4% paraformaldehyde and then observed under an optical microscope [35].

### 3.7. Inhibitory Effect of α-Amylase

The DNSA (3,5-dinitrosalicylic acid) technique was used to assess the alpha-amylase inhibition assay. The test combination was 500 µL of 0.02 M sodium phosphate buffer containing alpha-amylase (µg/mL) and AgNPs (10 to 10 µg/mL). The mixture was incubated at 37 °C for 20 min. At first, 250 µL of starch (1%) was introduced to the tubes and incubated at 37 °C for 15 min. The process was stopped by adding 1 mL of dinitrosalicylic acid, followed by incubating it for 10 min in a hot water bath. The tubes were cooled, and the absorbance at 540 nm was measured. The percentage of inhibition of α-amylase, which is shown in the equation below, was determined.

(4) Inhibition (%)=A−B/A×100

where A and B represent the absorbance of the control sample and the treated sample, respectively [5].

### 3.8. Inhibitory Effect of α-Glucosidase

The inhibition of α-glucosidase was determined by using the following method. The assay mixture contained 150 µL of sodium phosphate (0.1 M), alpha-glucosidase (1 U), and AgNPs (10–100 µg/mL). The mixture was preincubated at 37 °C for 10 min before adding paranitrophenyl alpha-D-glucopyranoside (50 µL, 2 MM) in sodium phosphate buffer (0.1 M) and incubating for 20 min at 37 °C. The reaction was stopped by adding sodium carbonate (50 µL, 0.1 M), and the absorbance at 405 nm was measured. The percentage inhibition was calculated using the following equation:

(5) Inhibition (%)=A−B/A×100

where A and B represent the absorbance of the control sample and the treated sample, respectively.

### 3.9. Antioxidant Activity

The DPPH (1,1-diphenyl-2-picryl hydrazyl) assay was used to determine the antioxidant activity of the synthesized AgNPs. The test samples and the standard were prepared at various concentrations (20, 40, 60, 100, and 200 g/mL) by adding ascorbic acid to 1 mL of 1 mM DPPH in ethanol and incubating for 30 min at 37 °C in the dark. The absorbance at 517 nm was measured using a UV–visible spectrophotometer. This mechanism involves the release of free radicals from DPPH, which the AgNPs scavenge, thereby inhibiting its antioxidant activity [36]. The standard formula to calculate the percentage of inhibition values is given in the below equation.

(6) Radical Scavenging=[Control−Test]/Test×100

## 4. Conclusions

The present study demonstrated the successful synthesis of AgNPs using *Rosa* *indica* L. petals extracted using ethanol, acetone, and water solvents. All three extracts produced spherical-shaped AgNPs; however, ethanol and acetone extracts produced smaller-sized AgNPs, while water extract produced larger-sized AgNPs. The ethanol and acetone extracts used to prepare AgNPs showed significant antibacterial, antidiabetic, and antioxidant activities. Furthermore, the overall results demonstrated that solvent-dependent extraction can preserve the bioactive molecules in the extract, indicating that the materials developed by these extracts can be favorable and can have the potential to be effectively useful in biomedical applications. Accordingly, the smaller-sized AgNPs were found to be excellent antibacterial agents for minimizing bacterial infections. Additionally, the biocompatibility results showed that the biosynthesized AgNPs could be used as potential candidates for various applications.

## Data Availability

Data is contained within the article.
